# Case Report: Insights into multiple cases of Pseudomyxoma peritonei

**DOI:** 10.3389/fgstr.2025.1529423

**Published:** 2025-03-31

**Authors:** HanJie Chu, XiaoBiao Song

**Affiliations:** ^1^ Baotou Clinical Medical College, Inner Mongolia Medical University, Baotou, China; ^2^ Department of Gastrointestinal Surgery, Baotou Central Hospital, Baotou, China

**Keywords:** Pseudomyxoma peritonei, heated intraperitoneal chemotherapy, cytoreductive surgery, mucinous, peritoneal neoplasms, case report

## Abstract

As a rare peritoneal malignant tumor, Pseudomyxoma peritonei (PMP) is a challenge in the field of surgery and oncology due to its complex pathological mechanism and difficult clinical management. Although the comprehensive treatment regimen centered on cell reduction (CRS) combined with intraperitoneal hyperthermic chemotherapy (HIPEC) has significantly improved the prognosis of patients, postoperative recurrence is still common, and repeated recurrence cases are particularly rare, and its clinical characteristics and treatment strategies need to be further explored. In this paper, the clinicopathological features, recurrence patterns and treatment difficulties of PMP were systematically analyzed by reviewing an elderly patient with three relapses and operation and combining with the data of four cases with different disease course. Through multi-case discussion and literature review, this study aims to provide clinicians with a more comprehensive understanding of the disease, optimize individualized treatment decisions, and provide references for further exploration of the molecular mechanism and novel therapeutic targets of PMP.

## 1 Introduction

PMP is a rare peritoneal malignancy characterized by the diffuse and progressive accumulation of mucinous, jelly-like sediments throughout the peritoneum and abdominal cavity. It is closely associated with appendiceal mucinous neoplasms and appendiceal perforation, and it may also secondarily predispose individuals to mucinous tumors in other abdominal organs (1, 2). Theories regarding the histomorphology, immunophenotype, and genetic alterations in the pathology of PMP are divergent, and the disease is currently the subject of significant international debate. This paper analyzes the common clinical and pathological features of PMP by focusing on one case and discussing it in conjunction with several similar cases.

## 2 Case description

The following cases were all from the General Surgery Department of Baotou Central Hospital

### 2.1 Case 1

**Figure 1:**
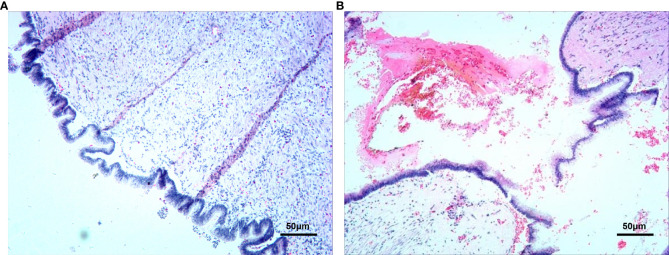
First operation, Pseudomyxoma of peritoneum Pathology. **(A, B)** (Stained by HE, At 200x).

A 63-year-old woman was admitted to the hospital on August 6, 2015, due to abdominal distension persisting for six months, accompanied by fatigue and poor appetite. The patient had a history of acute appendicitis perforation, which was successfully treated with conservative management for three weeks. Physical examination revealed a distended abdomen with an irregular, palpable mass in the middle and lower regions, measuring approximately 20cm*25cm*25cm. The mass exhibited excellent mobility, clear boundaries, and no tenderness, rebound tenderness, or muscle tension. Enhanced abdominal CT indicated a huge mass in the abdominopelvic cavity, suggestive of a cystadenoma. Upon review of the radiographs, a significant space-occupying effect and invasion of the suprahepatic space and omentum were noted. This raised the possibility of a ruptured appendiceal neoplasm adenoma with intraperitoneal implantation. Serum CEA levels were elevated at 26.47ng/m. Ultrasound-guided puncture of the anterior hepatic space yielded a jelly-like substance. The preoperative diagnosis was PMP. Peritoneoscopic investigation revealed a large amount of yellowish, jelly-like fluid in the abdominal cavity. The greater omentum was significantly thickened, hypertrophied, brittle, and hard in texture, presenting a cake-like shape and covered with cystic masses of varying sizes. The anterior hepatic space was filled with a jelly-like substance, and tiny nodules were scattered on the peritoneum. Adhesions involving the ileocecal region, appendix, and right ovarian adnexa formed a rigid mass. A large cystic mass, measuring approximately 25cm * 20cm * 20cm, was observed in the lower abdomen and pelvis, with a complete envelope that had ruptured at the top, allowing translucent jelly-like fluid to overflow from the sac. The intestinal surface was rough and irregular, with cysts of varying sizes present in the right and left paracolic sulci, splenic fossa, vesicouterine pouch, and rectouterine pouch. Surgical intervention involved the resection of the abdominal mass, right hemicolectomy, greater omentectomy, right adnexectomy, and abdominal drainage. Drainage catheters were placed in the right and left anterior hepatic spaces, the right subhepatic space, and the pelvis. Approximately 4000 ml of jelly-like fluid was aspirated intraoperatively. Postoperative pathological results revealed mucinous cystic adenocarcinoma in the right half of the colon, appendix, bilateral fallopian tubes, and ovaries. No cancer invasion was observed in the intestinal anastomosis margins, but cancer invasion was detected in all other tissues (**Figures 1A, B**). Postoperative heated intraperitoneal chemotherapy was administered using cisplatin and recombinant human endostatin, alternating weekly for a total of one month. The patient was discharged after the resolution of systemic symptoms and normalization of diet, urination, and defecation.

**Figure 2:**
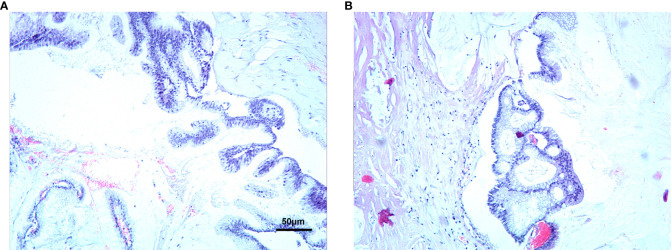
Second operation, Pseudomyxoma of peritoneum Pathology. **(A, B)** (Stained by HE, At 200x).

The patient returned to the hospital on June 12, 2018, due to the discovery of an abdominal mass persisting for 10 months. Examination revealed a 20 cm * 15 cm * 15 cm mass in the lower and middle abdomen, which was hard in texture, exhibited poor mobility, and had poorly defined boundaries. Serum CEA levels were 13.47ng/ml, and abdominal CT indicated the presence of PMP. Cytoreductive surgery was performed. Upon entering the abdomen through a median incision, a jelly-like cyst was found invading the abdominal wall, and the abdominal cavity was covered with a large amount of jelly-like fluid. Extensive growth of jelly was observed in all spaces, and numerous mucinous nodules of varying sizes were distributed in the intestinal wall and mesentery. Extensive mucinous cysts were present in the anterior hepatic space, splenic fossa, pelvis, peritoneum, and intestinal spaces. Adhesions were separated, and the mucinous cysts were extensively resected, yielding approximately 1000 ml of material. Approximately 2000 ml of intraperitoneal jelly-like fluid was suctioned out intraoperatively, and the abdominal cavity was treated with intraperitoneal spraying of 5-fluorouracil. Postoperative pathology confirmed mucinous adenocarcinoma in the abdominal cavity (**Figures 2A, B**).

The patient was admitted to the hospital again on March 22, 2021, due to an enlarged abdominal mass that had been present for six months. At this time, the mass had enlarged, and the patient experienced left-sided abdominal pain and discomfort. Multiple abdominal masses were detected on examination, with the largest measuring 15 cm in diameter. The masses were hard in texture, had limited mobility, and exhibited unclear boundaries. Serum CEA levels were 15.89 ng/ml. Enhanced abdominal CT revealed multiple cystic hypodense lesions in the abdominal and pelvic cavities, cysts in the lower part of the right lobe of the liver, multiple cysts in both kidneys, and a cystic lesion in the right interventricular area. PMP cytoreductive surgery, splenectomy, and partial transverse colectomy were performed. During the surgery, mucous jelly-like substance was observed in the abdominal wall, and the peritoneum was uneven with numerous protruding jelly-like cysts. The abdominal cavity was filled with a yellowish, transparent jelly-like substance. The jelly-like substance was removed, and intestinal adhesions were separated. A thick-walled cyst-like structure measuring 15 cm * 10 cm * 5 cm was observed in the left epigastrium. Upon incision of the anterior wall, a large amount of yellow, foul-smelling pus was discharged. The posterior wall of the abscess cavity was tightly adherent to the anterior wall of the stomach, while the anterior wall was surrounded by the transverse colon, descending colon, and splenic flexure of the colon, closely adherent to the intestinal wall. The upper edge of the abscess was closely adhered to the left lobe of the liver, and the abscess wall was approximately 5–6 mm thick. After suctioning the pus, the anterior wall was resected, revealing the abscess cavity. The orificium fistulae, with a diameter of about 1 cm, was found to be connected with the colon and exhibited a crater-like and hard texture. The intestinal canal in the abscess-affected section was stiff and thickened, necessitating resection. The close relationship between the spleen and cauda pancreatis, along with the bleeding and traumatic blood oozing, necessitated a splenectomy. The intestinal fistula was surgically repaired postoperatively. The postoperative pathological diagnosis identified mucinous adenocarcinoma of the colon in the specimen from the left half of the colon. The lesion was diffuse and extensive, invading the entire intestinal wall and extending beyond the plasma membrane with a perforation. Cancerous nodules were observed outside the intestinal wall, and the spleen was invaded. Some areas of the remaining intestinal wall were associated with suppurative inflammation. No cancer was observed in one side of the intestinal canal cut margin, while cancer was present in the other side. No cancer metastasis was observed in the mesenteric lymph nodes (0/5), but cancerous nodes were noted. Mucinous adenocarcinoma was observed in the wall of the abdominal abscess. The mucus-like material sent for examination from the abdominal cavity revealed no cellular component.

### 2.2 Case 2

A 53-year-old woman was admitted to the hospital on May 17, 2021, due to the discovery of a pelvic mass. Physical examination revealed a flat abdomen with no observable peristaltic waves in the gastral or intestinal pattern. The abdomen was soft, and the right lower abdomen appeared full, with a mass measuring 10 cm x 5 cm x 8 cm that had poorly defined boundaries and limited mobility. No obvious tenderness, rebound tenderness, or muscular tension was noted. Abdominal ultrasound indicated the presence of a cystic solid ma measuring approximately 15.4 cm * 4.5 cm in the ileocecal region of the right lower abdomen. Pelvic MRI revealed a huge cystic lesion in the right iliac fossa, measuring about 15.1 cm * 4.8 cm * 5 cm, with clear boundaries, a visible envelope, and compression of the affected adjoining intestinal canal. Enhanced abdominal CT demonstrated heterogeneous fatty liver and appendiceal mucus with diverticulum. The patient experienced recent anxiety and insomnia, without fever, dizziness, headache, or fatigue. She had lost 2.5 kg in the previous week and reported poor appetite and frequent urination. The patient had no relevant past medical history. The preoperative diagnosis was PMP. Intraoperative exploration revealed extensive adhesions of the colon to the omentum, lateral abdominal wall, and hepatic margin. The appendix was enlarged and thickened, with multiple elevated cystic nodules on the surface. Yellow jelly-like fluid was found around the appendix, in the pelvis, and between the small intestines, suggestive of an appendiceal neoplasm cystadenoma. Right hemicolectomy and partial peritonectomy were performed. The postoperative pathological report indicated mucinous cystadenomatous changes in the appendix, with significant fibrosis of the capsule wall and an absence of lymphoid follicle structures. Mild to moderate localized heterogeneous hyperplasia of the glandular epithelium was observed, with a pseudostratified arrangement of the nuclei. Focal capsule wall rupture was accompanied by cell-free mucus pools outside the capsule wall. These findings, in conjunction with the clinical presentation, were consistent with a low-grade appendiceal mucinous neoplasm (LAMN). Both large and small intestinal cut edges were clean, and no tumor involvement was observed in the omental tissue or any of the lymph nodes.

### 2.3 Case 3

A 50-year-old woman was admitted to the hospital on April 16, 2021, with a complaint of “right lower abdominal pain for 2 days.” Physical examination revealed a flat and soft abdomen, with no observable peristaltic waves of gastral or intestinal pattern. The right lower abdomen exhibited tenderness, without rebound tenderness or muscle tension. No mass was detected, and bowel sounds were slightly weak. Abdominal ultrasound indicated the presence of multiple uterine fibroids and a cystic mass in the right adnexal region, with effusions in the hepatic and renal spaces and the pelvis. The patient had no relevant past medical history. The preoperative diagnosis was PMP rupture and uterine fibroids. Intraoperative findings revealed a large amount of yellowish, jelly-like mucus in the abdominal cavity, some of which was removed. The appendix was found to be thickened, measuring approximately 5 cm in diameter and 7 cm in length, with jelly-like mucus emanating from the tail rupture. A portion of the appendiceal wall and mucus were sent for intraoperative pathology, which suggested malignancy. A broad ligament mass measuring about 4 cm x 5 cm x 5 cm was observed on the left side of the uterus, and jelly-like tissue attachment was noted on the surface of the uterus and bilateral adnexa. The patient’s mucinous tumor was considered malignant and had invaded the uterus and adnexa. Enlarged resection of the right colon and total hysterectomy with bilateral salpingo-oophorectomy were performed. Two pelvic drainage catheters were placed after the abdominal cavity was rinsed with distilled water. The postoperative pathological report confirmed the diagnosis of PMP. Multiple leiomyomas were found in the uterus and bilateral adnexa, along with endometrial proliferative phase changes and chronic inflammation of the cervix. One month after the surgery, the patient underwent two rounds of heated intraperitoneal chemotherapy. An abdominal CT scan performed three months postoperatively revealed no significant abnormalities.

### 2.4 Case 4

The hospital admitted a 49-year-old woman who complained of “abdominal pain for 2 days.” The patient developed right lower abdominal pain without obvious causes two days prior. This pain manifested as persistent distension and pain, accompanied by nausea and one episode of vomiting with gastric contents. Physical examination revealed a flat and soft abdomen, with positive tenderness in the lower abdomen, particularly in the right lower quadrant. This was accompanied by rebound tenderness and muscle tension, but no abdominal mass was detected. The preoperative diagnosis was acute appendicitis with peritonitis. Intraoperative exploration revealed thin, yellowish-white purulent fluid, approximately 20 ml, around the appendix and in the pelvis. The appendix measured approximately 5 cm in length and 1.3 cm in width, exhibiting cystic acute suppurative changes, thickening of the mesenteric edema, and adhesive wrapping of part of the omentum. A small amount of pus was present in the intestinal spaces. Laparoscopic appendectomy was performed, completely removing the yellowish-white pus from the abdominal and pelvic cavities, and a drainage catheter was placed in the pelvis. Postoperative pathological results indicated appendicular epithelial hyperplasia with mucinous cystadenoma changes.

### 2.5 Case 5

A 54-year-old woman was admitted to the hospital due to “distension and pain around the umbilicus and in the right lower abdomen for 10 days.” Ten days prior, the patient experienced right lower abdominal pain without obvious causes. This pain manifested as persistent distension and pain, with paroxysmal aggravation and periumbilical pain radiating to the lumbar back. The patient also experienced nausea and several episodes of vomiting, including gastric contents. Additionally, the patient had occasional cough and shortness of breath, as well as headache and sore throat, without fever or chills. The patient self-administered anti-inflammatory medication without significant relief and had no relevant past medical history. Physical examination revealed a flat and soft abdomen, with positive tenderness over the entire abdomen, particularly in the right lower quadrant. This was accompanied by rebound tenderness, without obvious muscle tension, and no abdominal mass was detected. An ultrasound of the lower right abdomen showed some cystic echoes in the upper right part of the ileocecal segment on the right side of the abdomen. This suggests that the patient may have an appendiceal mucous cyst or Meckel’s diverticulum. CT of the lower abdomen revealed a small amount of effusion in the pelvis, and colonoscopy demonstrated inflammatory changes in the transverse colon. The preoperative diagnosis was suspected PMP. Laparoscopic exploration revealed extensive and severe adhesions in the omentum and mesentery of the abdominal cavity. After adhesiolysis, adhesions and closure of the upper and lower interstitial spaces of the liver were observed. Exploration of the right lower abdomen after adhesiolysis revealed a cystic change in the appendix, measuring approximately 5 cm * 8.4 cm * 8.3 cm, with a smooth wall and high tension. The mesentery was congested with severe edema and thickening. Laparoscopic appendiceal mucinous cyst resection was performed, and the capsule wall was found to be intact in the samples. Upon opening the capsule wall, viscous, jelly-like cystic fluid was observed and sent for pathological examination. Pathological results after surgery showed that the appendix had chronic inflammatory changes, including blocking of the lumen, the loss of lymphoid follicles in the mucosa, and a lot of columnar and complexed mucus in the glandular epithelium. The nuclei displayed a pseudostratified arrangement, and the absence of cellular mucus outside the plasma membrane layer aligns with low-grade PMP changes and LAMN.

## 3 Discussion and reflection

### 3.1 Characteristics of PMP

PMP typically originates from lesions of the appendix, with fewer cases arising from ovarian lesions. The gelatinous substance diffuses further into the peritoneal space and peritoneal cavity (3) and implants in various organs of the abdominal cavity (2). Appendiceal mucinous neoplasms are the most common cause of PMP, with 20% of patients with these neoplasms developing PMP (1). PMP is a rare abdominal malignant tumor, with an incidence of approximately 1 to 3 cases per million per year (4), and is most frequently observed in women aged 50 years or older (5). The pathology of PMP is often indolent, and the disease is usually advanced at the time of detection. The cases described above are consistent with the clinical characteristics and development patterns of PMP. The clinical manifestations and physical signs of PMP are diverse (6), potentially including increased abdominal circumference, abdominal pain, and abdominal mass. The accumulation of mucous substances in the abdominal cavity compresses the organs, which may trigger manifestations such as intestinal obstruction, ascites, and nutritional disorders (7).

The World Health Organization (WHO) proposed a two-grade classification method in 2010, which deviates from the general oncological classification for tumors. Typically, mucin, cytology, and structural features classify PMP as low-grade or high-grade (low-grade appendiceal mucinous neoplasm [LAMN]/high-grade appendiceal mucinous neoplasm [HAMN]). This classification serves as an important tool for predicting tumor behavior and guiding treatment strategies (8). PMP typically expresses immunohistochemical markers, such as CK20, CDX2, and MUC2, positively, and these markers are associated with KRAS mutations and deletions at specific gene loci (3).

### 3.2 Relevant examinations and assessments

The condition of PMP correlates with serum tumor markers, as demonstrated in several reports. Tumor load in PMP patients relates to CEA and CA19-9 levels, and high-level expression of CA19-9 correlates with a high recurrence rate. The degree of CEA expression at the time of PMP recurrence is associated with prognosis. Generally, prognostic predictors of PMP include CA125, CA19-9, and CEA (9, 10), and preoperative elevation of these three tumor markers reduces overall survival and disease-free survival (4).

Abdominal and pelvic CT scans are the most commonly used imaging examinations for PMP and can detect appendiceal mucinous cysts in the early stages of the disease (4). However, MRI and PET-CT have limitations in the diagnosis of PMP (9). Preoperative imaging scoring using MDCT can predict the resectability and survival rate of patients with PMP by measuring the tumor load in the perihepatic region (4).

### 3.3 Treatment principles

The standard treatment method for PMP currently combines CRS with HIPEC (11). In the treatment of PMP with intraperitoneal HIPEC, the ideal chemotherapy agent should possess three characteristics: effective tissue penetration, uniform distribution in the peritoneal cavity and blood, and slow peritoneal absorption rate. Drugs commonly used for intraperitoneal administration in clinical practice include mitomycin C, oxaliplatin, irinotecan, paclitaxel, docetaxel, and doxorubicin. However, the application of antimetabolic drugs such as 5-fluorouracil and gemcitabine in HIPEC therapy is limited due to the continuous action on the tumor surface. It is worth noting that various chemotherapy drugs in the existing HIPEC drug system present unique therapeutic advantages and clinical application limitations in the treatment of PMP.However, PMP still frequently recurs even with this treatment approach (4). Patients should have regular abdominal and pelvic CT reviews after CRS and HIPEC are completed to prevent PMP recurrence.If recurrence occurs, intervention and treatment should be undertaken as soon as possible. If surgical treatment is not suitable for the patient or CRS combined with HIPEC cannot be performed, neoadjuvant chemotherapy can be used to help reduce tumor cells (12).

### 3.4 Latest research results and breakthrough points

Recent research suggests that microorganisms may play a role in the pathogenesis of PMP, prompting a few studies targeting Helicobacter pylori in PMP. However, a pronounced correlation requires more reliable evidence-based medical evidence (13).

It was found by Kjersti Flatmark et al. that GNAS mutations were present in PMP tumor samples and that GSα peptides have a high level of immunogenicity. This finding suggests that GSα peptides may be used to reinforce pre-existing immunity in patients with PMP and induce *de novo* immunity to the mutant Gsα. This treatment strategy may offer therapeutic possibilities for patients with PMP and other cancers with GNAS mutations (14).

## 4 Summary and reflection

Collectively, based on the above cases and studies, it is evident that PMP is a rare peritoneal malignant tumor closely associated with appendiceal mucinous neoplasms and appendiceal perforation. A small proportion of PMP cases originate from other tissues and organs, such as the ovary. CT is the most important imaging tool for PMP. By using imaging to detect appendiceal lesions in a timely manner, it may be possible to avoid the development of appendiceal lesions into PMP through early surgical intervention before appendiceal perforation and cyst rupture occur. We hypothesize that the scope of surgery should encompass not only the appendiceal tissue but also the peritoneum and surrounding organs, taking into account the existing international treatment guidelines and the cases described above. Attempting to remove as much disseminated mucus as possible may have a positive effect on prognosis. Serum tumor markers, CA125, CA19-9, and CEA, are prognostic predictors of PMP and they are associated with overall survival and disease-free survival.

CRS combined with HIPEC is the standard clinical treatment for PMP, while neoadjuvant chemotherapy has a tumor cell-reducing effect in patients who are not suitable for surgery. We will conduct further follow-up visits with the patients in the presented cases and adopt appropriate adjuvant treatments to prolong their survival time.

## Data Availability

The datasets presented in this study can be found in online repositories. The names of the repository/repositories and accession number(s) can be found in the article/supplementary material.
